# Effects and mechanisms of vitamins A and D on behavior associated with Tourette syndrome in rats

**DOI:** 10.3389/fnut.2025.1561693

**Published:** 2025-09-15

**Authors:** Li-Na Li, Ying Xu, Xiang-Jun He, Wen-Xin Zhang, Hui-Feng Zhang, Li Song, Hai-Shui Shi, Xiao-Yu Tian, Xue Yan

**Affiliations:** ^1^The Department of Pediatrics, The Second Hospital of Hebei Medical University, Shijiazhuang, China; ^2^Neuroscience Research Center, Institute of Medical and Health Science, Hebei Medical University, Shijiazhuang, China; ^3^Hebei Key Laboratory of Early Life Health Promotion (SZX202419), Hebei Medical University, Shijiazhuang, China; ^4^Nursing School, Hebei Medical University, Shijiazhuang, China; ^5^Hebei Key Laboratory of Neurophysiology, Hebei Medicinal University, Shijiazhuang, China

**Keywords:** Tourette syndrome, vitamin A, vitamin D, gut microbiota, metabolites

## Abstract

**Background and aims:**

Tourette syndrome (TS) is a complex neurodevelopmental disorder involving genetic, neurotransmission, immune, and gut–brain axis mechanisms that cause persistent motor and vocal tics. Current treatments mainly include drugs (for example, neuroleptics), but the treatment strategies are often unsatisfactory. Retinoic acid, derived from vitamin A, plays a role in TS, as children with this condition often exhibit reduced serum levels of retinoic acid and 25-hydroxyvitamin D. Supplementing with either nutrient has been shown to alleviate tic severity and frequency. In this study, we aim to explore the therapeutic effects and possible mechanisms of vitamins A and D in TS.

**Methods:**

3,3-Iminodipropionitrile (IDPN) was used to induce acute TS-like symptoms in rats via striatal dopamine dysregulation. Then vitamins A (3 mg/kg/d) and D (10ug/kg/d) were continuously administered by gavage for 8 weeks. Stereotyped and head twitching behavior tests were then performed. High-performance liquid chromatography was used to determine serum levels of 25-OH-D3, calcium, phosphorus, and alkaline phosphatase in rats. The composition of intestinal flora was analyzed using 16S rRNA gene sequencing, and striatum metabolites were detected by untargeted metabolomics.

**Results:**

The experimental results showed that the tic phenotype of TS rats was significantly relieved after 8 weeks of vitamin A and D treatment (*p* < 0.001, *n* = 10). The blood vitamin content and neurotransmitters in the striatum of rats changed after vitamin A and D treatment (*p* < 0.05, *n* = 6). Moreover, vitamins A and D caused obvious changes in the diversity of intestinal flora and the abundance of each species in TS rats.

**Conclusion:**

This study shows that vitamin A and D supplementation can significantly alleviate the tic phenotype of TS rats, which demonstrates an association between vitamin A/D-induced gut flora alterations and neurotransmitter changes. Further research is needed to establish direct causal relationships in the gut–brain axis pathway.

## 1 Introduction

Tourette syndrome (TS) is a common pediatric disease, and the core clinical manifestations of TS consist of multiple motor tics and at least one vocal tic, which commonly present with psychiatric comorbidities, particularly obsessive-compulsive disorder (OCD) and attention deficit hyperactivity disorder (ADHD). TS affects 0.3–1% of the population, typically emerging in childhood. While currently incurable, symptoms can be effectively managed, with only 5–10% of cases persisting throughout adulthood (1). At the onset of the disease, children may have symptoms such as blinking, frowning, and shaking their heads, which seriously affect their quality of life and social ability. Currently, there is no definitive curative treatment for TS in clinical practice. Commonly used drugs such as neuroleptics for the treatment of TS in children all have different degrees of adverse drug reactions (such as dizziness and lethargy), which significantly limit their clinical application. A drug combined with psychological behavior therapy is a commonly used clinical treatment method (2). Comprehensive Behavioral Intervention for Tics (CBIT), the first-line psychological therapy for TS, includes habit reversal training and functional interventions to reduce tic severity and improve coping (3).

It is considered that TS results from the combination of multiple predisposing genes and the influence of environmental factors (4). Among the environmental factors, micronutrients, such as vitamins A and D, are of interest to many scientists. Vitamin D (VD) is a neurosteroid hormone that plays in neurodevelopment, immune function, and bone health. VD can regulate the expression of more than 200 genes, protecting the dopamine system through gene expression regulation. VD receptor (VDR) and its activating enzyme 1α-hydroxylase are widely present in the human brain. Studies have suggested that they have neurotrophic and neuroprotective properties, affecting neuroplasticity, neurotransmission, and neuroimmunity (5–7). Vitamin A (VA) is also an important micronutrient, and its active form, retinoic acid (RA), is closely related to cell development and differentiation in the central nervous system. Furthermore, VA and VD synergistically regulate gene expression, and VD and RA interact with VD receptor and retinoic acid receptor complexes in the nucleus to further regulate gene expression. VA and VD deficiencies may contribute to TS pathogenesis through distinct mechanisms (8). VA regulates striatal dopamine receptor expression via retinoic acid signaling, while VD modulates neuroinflammation and synaptic plasticity—both pathways implicated in TS neurobiology (9, 10). Clinical studies have found that children with tic disorder show reduced serum 25-hydroxy-vitamin D [25(OH)D] and retinoic acid levels, and their tic symptoms are relieved after VA or VD supplementation (11, 12). However, there are no specific studies on the combination therapy and the possible mechanism of vitamin A and D supplements for tic disorder. Unlike Parkinson’s disease, which is characterized by nigral neurodegeneration, TS pathophysiology involves functional alterations in striatal dopamine signaling without neuronal loss. Dopamine (DA) is a neurotransmitter released by nerve endings of midbrain neurons. It masters all aspects of nerve function (motor, emotion, sleep, etc.), and is involved in the activation of the dorsolateral prefrontal cortex, striatum, and thalamus, and the regulation of cortical connections with the basal ganglia. In the basal ganglia, dopamine D2 receptors (D2Rs) are abundantly expressed in medium spiny neurons (MSNs) and dopaminergic neurons themselves, serving as pivotal regulators of dopaminergic signaling. Clinical evidence demonstrates that D2R-targeting agents effectively alleviate TS symptoms, confirming that the modulation of striatal dopaminergic signaling—particularly D2R function—represents a core therapeutic strategy for TS (13). Notably, certain nutritional factors, particularly deficiencies in vitamin A (VA) and vitamin D (VD), may disrupt neuromuscular function by impairing calcium signaling and neurotransmitter homeostasis, potentially contributing to the pathogenesis of specific disorders (14). There is substantial evidence that vitamins can eliminate abnormal dopamine and calcium signaling, especially VD, and the interaction of VA and VD can improve movement disorders. In addition, retinoic acid can regulate the mRNA expression of D2Rs by interacting with retinoic acid receptors, thereby affecting the DA signaling pathway and subsequently affecting nerve function. VD deficiency easily leads to significant changes in serotonin and dopamine levels, as well as decreased levels of glutamate and glutamine, which in turn affect neuromuscular conduction (15).

In recent years, studies have shown that intestinal microecology is closely related to the occurrence of TS. Specifically, microbial metabolites can modulate neuroinflammation and dopaminergic activity in cortico-striatal circuits, while bacterial overgrowth may trigger immune responses that exacerbate TS (16, 17). Human intestinal microorganisms play a significant role in various critical physiological processes such as metabolism, nutrient digestion, and immune regulation. Their quantity and diversity eventually stabilize after the developmental transition stage in infants and young children, resembling the quantity and structure of adult flora (18). The microbe–gut–brain axis is a bidirectional communication system in which intestinal flora regulates the central nervous system through neurotransmitters, immune responses, the endocrine system, and metabolites (19). Related studies have shown that the structure and abundance of intestinal flora in children with TS differ significantly from those in healthy children. The relative abundance of harmful bacteria, such as Turicibacteraceae and Ruminococcaceae, increases significantly, and the symptoms of TS improve notably after fecal transplantation (20, 21). Studies have reported that the gut–brain axis is involved in the occurrence and development of TS. VA deficiency can alter the function of the gut microbiota, while VD regulates the gut microbiome by inhibiting inflammation, maintaining barrier function, and promoting microbial homeostasis (22). As an essential component of the immune system, the intestinal mucosal barrier prevents the invasion of harmful substances and maintains internal homeostasis. VD influences the structure and diversity of the gut microbiome through VD and VDR signal transduction pathways, regulates the proliferation and differentiation of intestinal immune cells, affects the tight junctions of intestinal epithelial cells, and modulates immune factors vital for mucosal immunity (23). In this study, VA and VD were administered via gavage to TS rats, followed by behavioral tests and the collection of blood, brain tissue, and stool samples, to explore the therapeutic effects of VA and VD on TS and their potential mechanisms.

## 2 Materials and methods

### 2.1 Laboratory animals

Fifty male 3-week-old SD rats were purchased from Beijing Sbeifu Biotechnology Co., Ltd. and kept in a specific pathogen-free (SPF) room of the Experimental Animal Center of Hebei Medical University under environmental control temperature (23 ± 3 °C), relative humidity 50 ± 10%, light/dark cycle for 12 h, with free access to food and water. This study was conducted in accordance with the recommendations of the Guidelines for the Care and Use of Laboratory Animals of the Chinese Institute of Health. The ethics committee of Hebei Medical University approved all animal experiments.

### 2.2 Animal grouping, IDPN treatment, VA, and VD gavage

After 1 week of adaptation to the new environment, the rats were randomly divided into the control group (*n* = 10) and the TS group (*n* = 40). The TS model was established through the systemic administration of the neurotoxin IDPN (3,3′-iminodipropionitrile), which induces characteristic tic-like behaviors, including prominent head twitching (HTR) and stereotyped circling via dopaminergic dysregulation in cortico-striatal circuits. The rats in the control group were intraperitoneally injected with normal saline (1 mL/kg/d), and the Model group was intraperitoneally injected with IDPN (150 mg/kg/d) for 7 consecutive days. Subsequently, rats in the TS group were further randomly assigned to Model (TS), VA (TS + VA), VD (TS + VD), and VA + VD (TS + VA + VD) groups (*n* = 10). The VA group was given vitamin A dissolved in corn oil (1 mL/kg/d) at a dose of 3 mg/kg/d for 8 weeks, the VD group was given vitamin D3 dissolved in corn oil at a dose of 10ug/kg/D for 8 weeks, and the VA + VD group was given vitamins A and D at the same dose for 8 weeks. Model and Con were given corn oil for 8 weeks.

### 2.3 Behavioral approaches

Behavioral tests were conducted in an opaque open box (35 cm length × 25 cm width × 25 cm height). The equipment was cleaned before and after each experiment to maintain a clean and odor-free environment. Rats were transferred to dedicated behavioral laboratories in advance and allowed to acclimate for at least 3 h to reduce stress. Animals were gently removed from their cages and placed in the apparatus with their backs facing the experimenter. The experimenter then quickly and quietly left. Two blinded experimenters recorded stereotyped behaviors (in-place circling) and head twitching behaviors (HTR) for 15 min. After testing, the rats were returned to their cages, labeled, and the apparatus was cleaned with alcohol and paper towels.

### 2.4 Experimental animal materials

#### 2.4.1 Feces samples

After anesthesia with 2% sodium pentobarbital, a midline laparotomy was performed. The intestines were exposed, and 500 mg of the cecal content was collected and stored at −80 °C.

#### 2.4.2 Serum samples

Following anesthesia, rats were placed in a supine position. The chest area was shaved and disinfected. A syringe with a 4–5-gage needle was used to puncture the third–fourth left intercostal space at the heart’s strongest beat. Approximately 2 mL of blood was collected and centrifuged. The serum was stored at −80 °C.

#### 2.4.3 Striatum samples

After blood collection, the rat was decapitated. The scalp was opened, the skull was carefully removed, and the cerebral cortex was lifted to expose the striatum, located above the hippocampus. The striatum was isolated, placed into a centrifuge tube on ice, flash-frozen in liquid nitrogen, and stored at −80 °C.

### 2.5 High-performance liquid chromatography–tandem mass spectrometry (HPLC-MS/MS)

Serum 25-OH-D3, calcium (CA), phosphorus (P), and alkaline phosphatase (ALP) were detected using a liquid chromatography tandem mass spectrometer (4500MD) of AB SCIEX Company, USA. The pretreatment method of liquid–liquid extraction is adopted to extract the test substance from the serum to the greatest extent. It completely separates the substance to be measured from impurities by liquid chromatography, and further purifies and separates the substance to be measured. Finally, tandem mass spectrometry was used for qualitative and quantitative analyses.

### 2.6 16S rRNA diversity sequencing experimental methods

DNA extraction and PCR amplification using the MagPure Soil DNA LQ Kit (Magan). The kit performs the extraction of genomic DNA from the intestinal contents sample according to the instructions. The concentration and purity of the DNA were measured using a NanoDrop 2000 (Thermo Fisher Scientific, USA) and agarose gel electrophoresis. The extracted DNA was then stored at – 20 °C. PCR amplification of the bacterial 16S rRNA gene was performed using specific primers with a Barcode and Takara Ex Taq high-fidelity enzyme, with the extracted genomic DNA as a template. The 16S rRNA gene was amplified using universal primers 343F (5-TACGGRAGGCAGCAG-3) and 798R (5-AGGGTATCTAATCCT-3) and 515F (5-GTGCCAGCMGCCGCGG-3) and 907R (5-CCGTCAATTCMTTTRAGTTT-3) V3–V4 (or V4–V5) variable regions for bacterial diversity analysis. Library construction and sequencing PCR amplification products were detected using agarose gel electrophoresis. Then, AMPure XP magnetic beads were used for purification, which was used as a second-round PCR template after purification, and a second-round PCR amplification was performed. Again, using magnetic beads to purify, the purified second-round product was used for Qubit quantification, and then the concentration was adjusted for sequencing. Sequencing was performed using an Illumina NovaSeq 6000 sequencing platform, and 250 bp double-ended reads were generated. Sequencing was performed by Shanghai Ouyi Biotechnology Co., Ltd. (Shanghai, China).

### 2.7 16S rRNA diversity sequencing analysis methods

DNA extraction from fecal samples was performed using the MagPure Soil DNA LQ Kit (Magan), following the manufacturer’s protocol. DNA concentration and purity were assessed using a NanoDrop 2000 (Thermo Fisher Scientific, USA) and agarose gel electrophoresis. Extracted DNA was stored at −20 °C. Bacterial 16S rRNA genes were amplified using specific primers with barcodes and Takara Ex Taq high-fidelity enzyme. Primers 343F (5-TACGGRAGGCAGCAG-3), 798R (5-AGGGTATCTAATCCT-3), 515F (5-GTGCCAGCMGCCGCGG-3), and 907R (5-CCGTCAATTCMTTTRAGTTT-3) were used to target the V3–V4 (or V4–V5) regions. Amplified products were purified using AMPure XP magnetic beads, followed by a second PCR amplification and purification. *Metabolites were annotated using the HMDB database (version 5.0).* Final products were quantified using Qubit, adjusted to the appropriate concentrations, and sequenced on the Illumina NovaSeq 6,000 platform to generate 250 bp paired-end reads. Sequencing was conducted by Shanghai Ouyi Biotechnology Co., Ltd. (Shanghai, China).

### 2.8 Image processing and statistical analysis

Identified metabolites were functionally annotated (HMDB/KEGG) and grouped by biological roles: (1) synaptic phospholipids (PS/PC) maintaining neuronal membrane integrity, (2) neuroactive microbial-host cometabolites (e.g., anti-inflammatory trans-cinnamic acid), and (3) microbial butyrate derivatives (e.g., butyramide). All data are presented as mean ± SEM. Analyses were performed using SPSS 22.0 software (IBM Corporation, Armonk, NY, USA) and GraphPad Prism version 7.0 statistical software (GraphPad, Inc., La Jolla, USA). Statistical analyses were performed using one-way analysis of variance (ANOVA) and Bonferroni *post hoc* tests (for normal distribution and variance homogenization data), as well as non-parametric tests (for other data). *p*-values of <0.05 were considered statistically significant.

## 3 Results

### 3.1 IDPN induces tic-like behavior in SD rats

**Figure 1:**
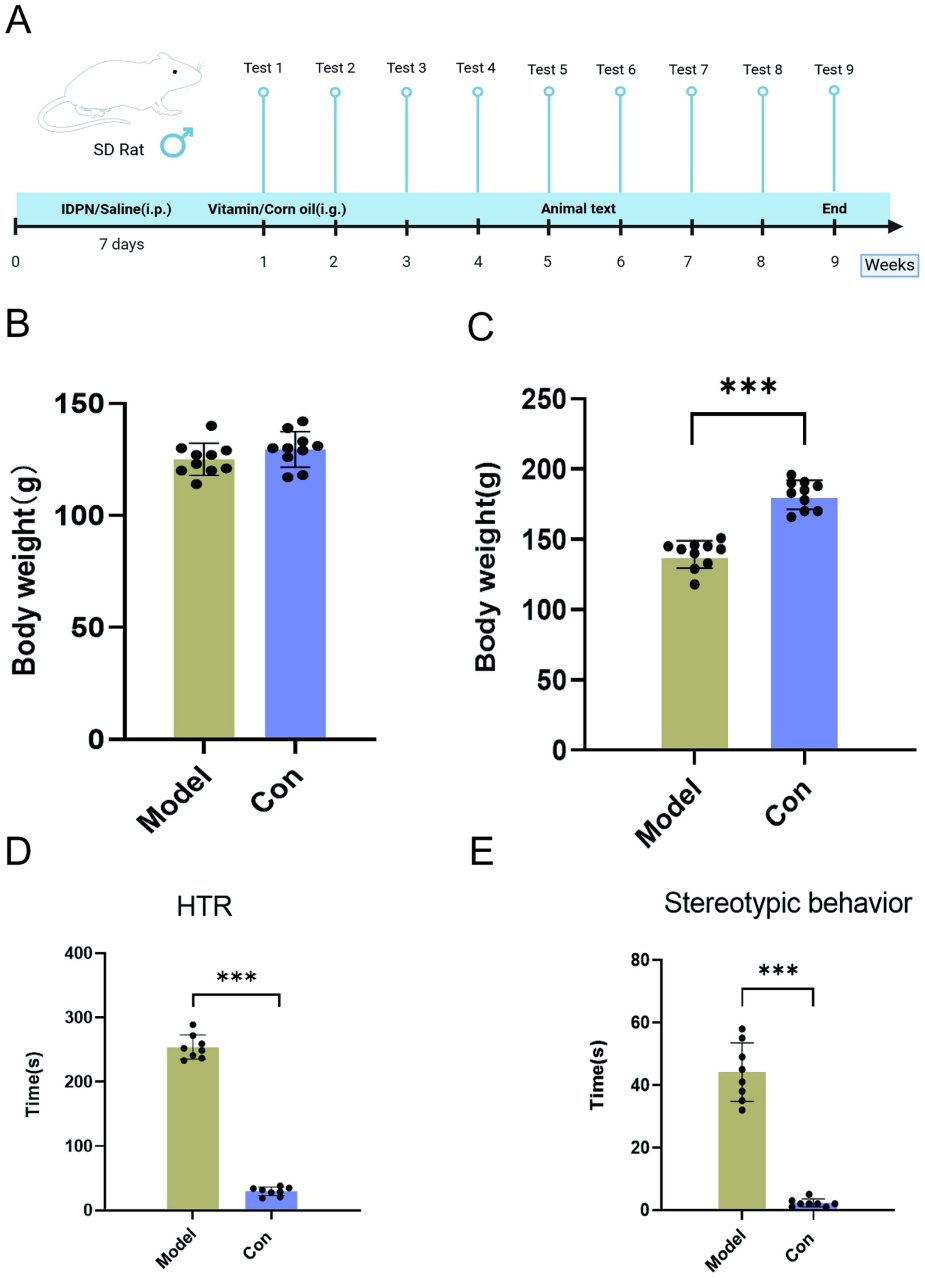
Experimental schedule of experimental procedures, IDPN and vitamin A, D treatment, testing, and sample collection **(A)**. Behavioral tests and weighing were performed weekly. Serum, striatum, and fecal samples were collected on the last day of vitamin tube feeding. Mice were killed 2 days after the last tube feeding. Body weight changes before **(B)** and after modeling **(C)**. HTR **(D)** and stereotyped behavior **(E)** changes before and after modeling. (Values are means ± SEMs; different from control, (*n* = 8) ***p < 0.01).

The TS model was established by the systemic neurotoxin administration method created by Diamond et al. (24). After IDPN administration, behaviors such as head twitching behavior (HTR), circling *in situ* (stereotyped behavior), and licking the front paw occurred. Among them, head twitching and stereotyped behavior were obvious, easily distinguishable, and the frequency of twitching was high. As a criterion for the success of the model, the behavior of the rats was assessed on the seventh day of modeling (Figure 1A). Compared with the control group, the model group rats showed a significant increase in HTR and stereotypic behaviors, as well as weight loss, indicating successful model establishment (Figure 1B–E). These behavioral changes in the rats of the Model group were similar to the clinical manifestations of human tic disorder, indicating the high reliability of the IDPN-induced TS model.

### 3.2 VA and VD improve TS behaviors, growth, and development in rats

**Figure 2:**
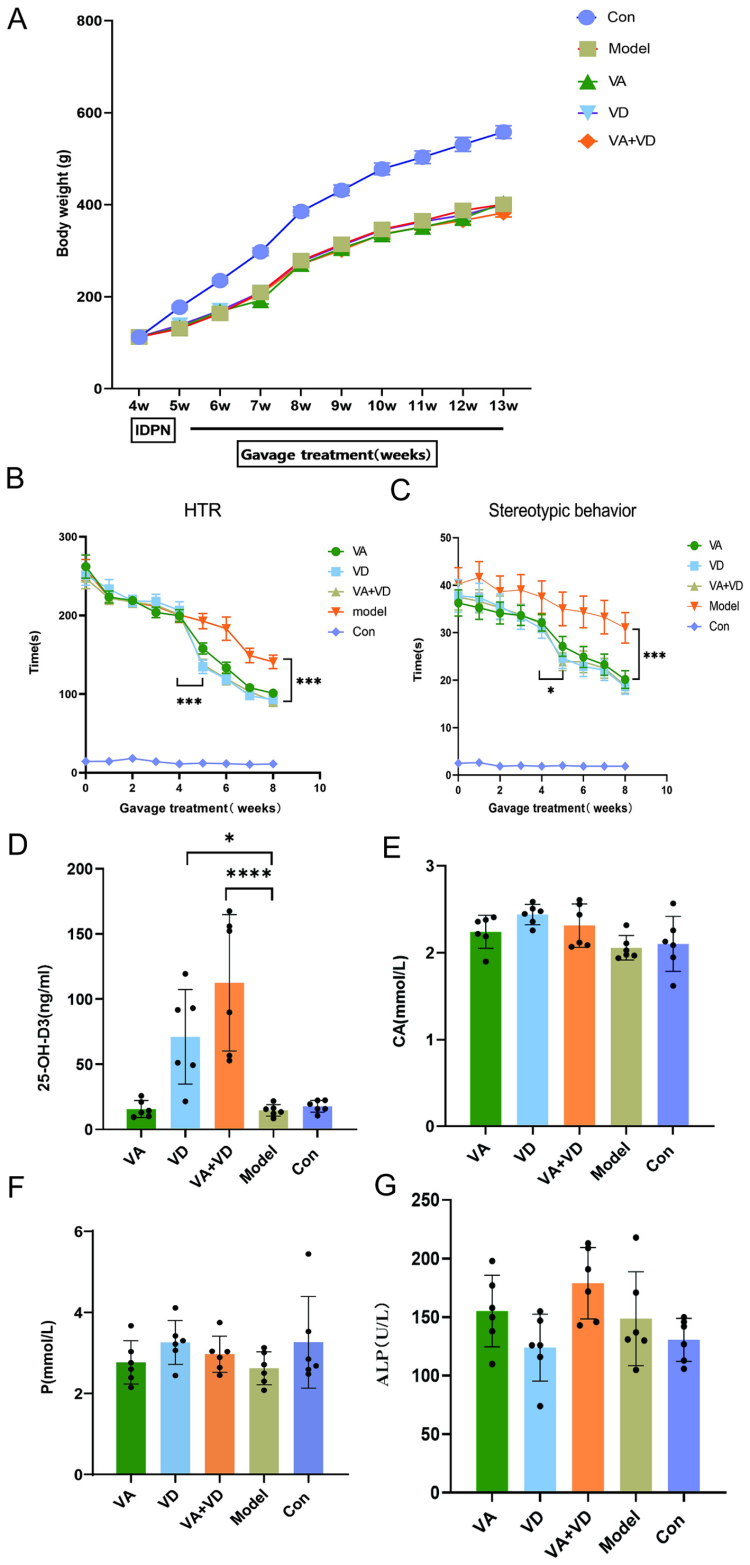
Body weight change **(A)**, HTR **(B)**, and stereotypic behavior change **(C)** from model creation to material extraction in each group; (*n* = 8) the contents of serum 25-OH-D3, CA, P, ALP **(D–G)** in each group.(Values are means ± SEMs; different from control, (*n* = 6)*p < 0.05, **p<0.01, ***p < 0.001, ****p < 0.0001).

The supplementation of vitamins A and D significantly reduced the frequency and intensity of tics in TS rats. Compared with the Model group, the HTR and stereotypic behavior in the VA, VD, and VA + VD groups were significantly reduced, but the VD and VA + VD groups showed more significant differences than the VA group (*p* < 0.001, Figures 2B,C). Among the treatment groups, the weight of rats in the VA, VD, and VA + VD groups increased; however, there was no statistically significant difference compared with the Model group (Figure 2A).

### 3.3 Serum 25-OH-D3 levels increased after vitamin treatment

After modeling, the Model group showed a decreasing trend in 25-OH-D3 levels compared to the Con group, but there was no statistically significant difference. After 8 weeks of vitamin treatment, the serum 25-OH-D3 levels increased in both the VD and VA + VD groups, with the VA + VD group showing significantly higher serum vitamin D levels than the VD group (Figure 2D). There were no statistical differences in the content of CA, P, and ALP in the serum among the Model, Con, VA, VD, and VA + VD groups (Figures 2E–G).

## 4 Effects of VA and VD on differential metabolites

### 4.1 Multivariate data analysis of striatal metabolites

**Figure 3:**
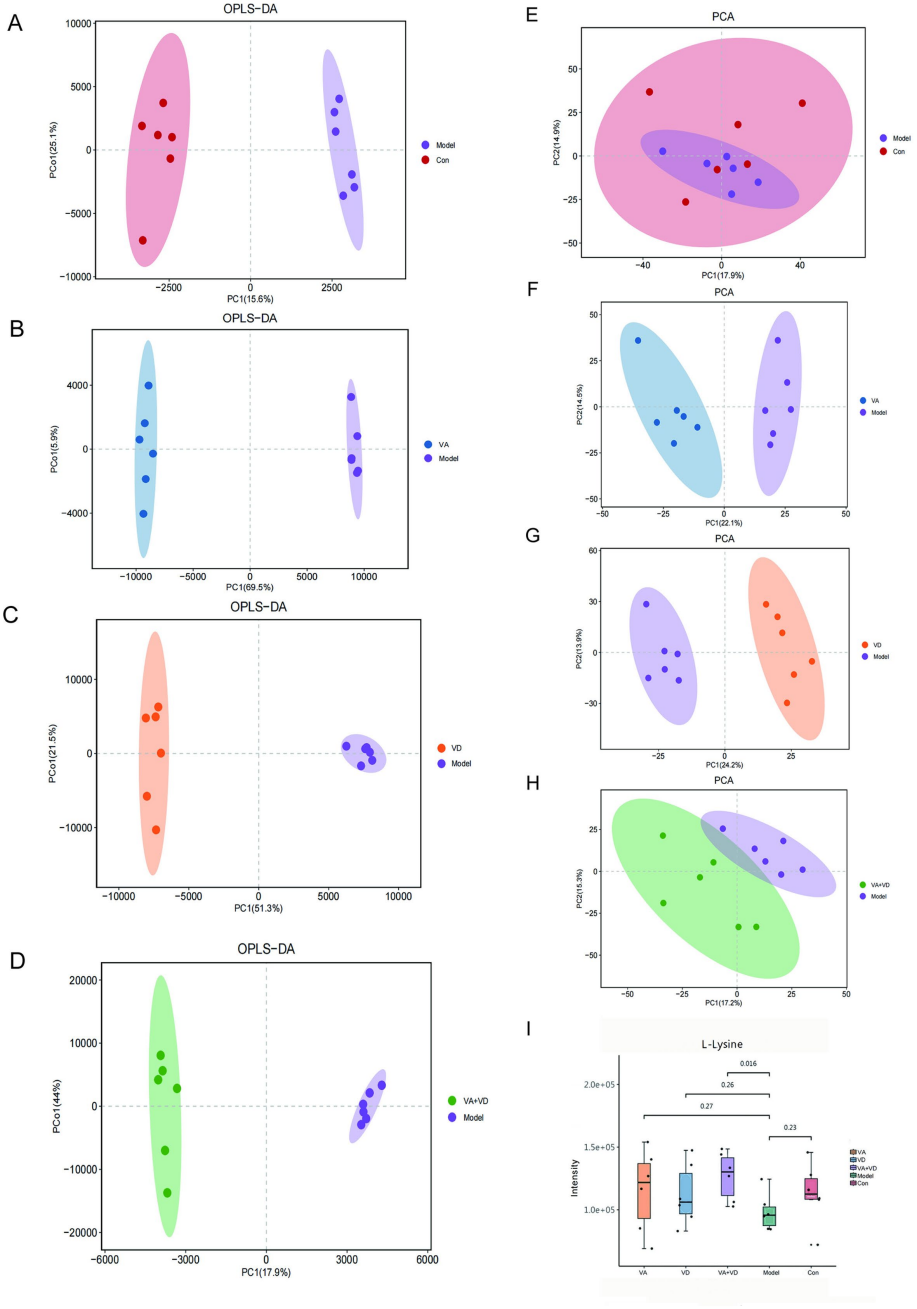
Vitamins A and D significantly regulate the striatum metabolic profile of TS rats. OPLS-DA score **(A–D)** and PCA score **(E–H)** for each group; serum differential metabolite L-lysine change **(I)**. (Values are means ± SEMs; *n* = 6).

We used OPLS-DA in supervised mode to analyze data from the VA, VD, VA + VD, Model group, and Con groups to investigate the effects of vitamins A and D on the metabolic pattern of TS rats. A clear separation was observed between the model and control groups (Figure 3A), suggesting that tics exerted a significant effect on the composition of brain tissue metabolites. The samples in the VA, VD, and VA + VD groups differed from those in the Model group, respectively (Figures 3B–D). The separation between the groups indicated that the metabolites in the groups were significantly different. This result confirms the role of VA and VD treatment in ameliorating brain injury by modulating certain metabolic pathways.

### 4.2 Identification of potential endogenous differential metabolites

Analysis of the striatum metabolite composition showed that in the Model and Con groups, a total of 22 significantly different metabolites were identified (Table 1), including a variety of fatty acids, amino acids, and depsipeptides, some of which are closely related to nervous system function. Substances closely related to the development of the nervous system, such as L-lysine, which plays an important role in neurodevelopmental disorders (25), have shown significant changes. Compared with the control group, the L-lysine content in the striatum of rats in the model group decreased, and the content in the vitamin group increased (Figure 3I). PCA was used to reflect metabolite variability between and within groups. We observed differences in the types and relative abundances of metabolites between the control and model groups, the VA and model groups, the VD and model groups, and the VA + VD and model groups (Figures 3E–H). As shown in the figures, significant differences between the VA, VD, VA + VD groups, and the Model group are shown in the metabolome.

**Table 1:** The identification of differential metabolites between Model and Con group.

NO.	Ion mode	HMDB	Metabolites	Model / Con	
				VIP	Trend
A1	pos	HMDB0008039	PC(18:0/18:2(9Z,12Z))	24.449	↑∗∗
A2	pos	HMDB0010167	PS(18:0/22:6(4Z,7Z,10Z,13Z,16Z,19Z))	11.452	↑∗∗∗
A3	neg		PC(15:0/19:1(9Z))	8.461	↑∗
A4	neg	HMDB0012444	PS(22:6(4Z,7Z,10Z,13Z,16Z,19Z)/18:0)	7.369	↑∗∗∗
A5	pos	HMDB0033870	Butyramide	5.91	↓∗∗
A6	pos	HMDB0012382	PS(18:0/20:3(8Z,11Z,14Z))	5.168	↑∗∗∗
A7	neg		PC(15:0/17:0)	4.512	↑∗
A8	neg	HMDB0286444	PC(6 keto-PGF1alpha/18:1(9Z))	4.457	↑∗
A9	pos	HMDB0282329	PS(20:2(11Z,14Z)/22:6(5Z,7Z,10Z,13Z,16Z,19Z)-OH(4))	3.191	↑∗∗∗
A10	pos	HMDB0008090	PC(18:1(11Z)/22:6(4Z,7Z,10Z,13Z,16Z,19Z))	3.019	↓∗∗
A11	neg	HMDB0112617	PS(20:3(5Z,8Z,11Z)/22:5(4Z,7Z,10Z,13Z,16Z))	3.016	↓∗∗
A12	pos	HMDB0000930	Trans-Cinnamic Acid	2.579	↓∗
A13	pos	HMDB0301824	Isoketocamphoric Acid	2.502	↑∗
A14	neg		Dolastatin 16	2.264	↑∗
A15	neg		PE(O-18:1(9Z)/0:0)	1.966	↑∗
A16	pos	HMDB0248106	Alanine Pyruvate	1.836	↑∗
A17	pos	HMDB0248220	Alpha-Hydroxy-Beta-Phenylethylamine	1.635	↓∗
A18	pos	HMDB0000182	L-Lysine	1.278	↓#
A19	pos	HMDB0006581	3’-N-Acetylneuraminyl-N-Acetyllactosamine	1.251	↑∗
A20	neg		Nystatin	1.159	↑∗∗
A21	pos	HMDB0255163	N-Isopropylacrylamide	1.074	↑∗
A22	pos	HMDB0033479	Sinapoylspermine	1.005	↑∗∗∗

↑, up-regulated; ↓, down-regulated; *p < 0.05, *p* < 0.01, *p < 0.001, #*p* < 1.

### 4.3 Analysis of related metabolic pathways and targets

**Figure 4:**
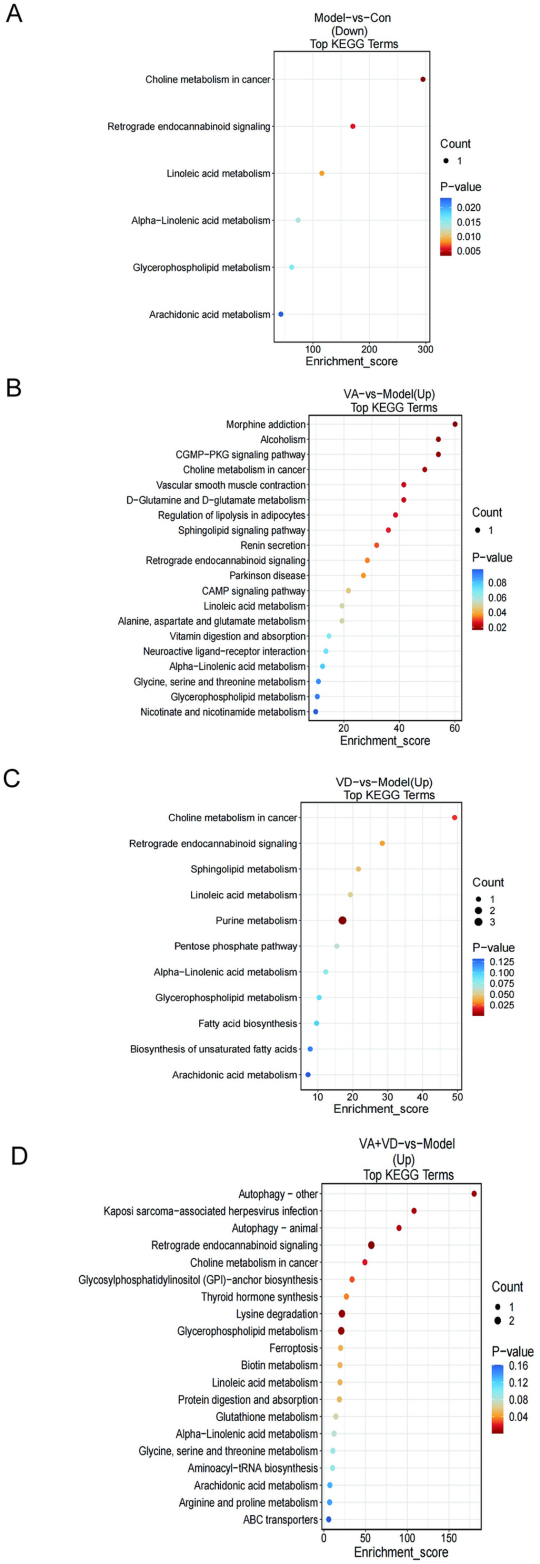
Vitamins A and D affect the striatal metabolic pathway in TS rats. **(A)** Metabolic pathways analysis between the Model and Con, **(B)** VA and Model, **(C)** VD and Model, and **(D)** VA + VD and Model. (Values are means ± SEMs; *n* = 6, *p*<0.05).

We performed metabolic pathway enrichment analysis of striatum differential metabolites in the control and Model groups, the VA + VD and Model groups, the VA and Model groups, the VD and Model groups based on the KEEG database, and obtained the KEEG bubble diagram (Figures 4A–D). As shown in the figure, the signal transduction pathways such as retrograde endocannabinoid signaling, glycerophospholipid metabolism, and alpha-linoleic acid metabolism in the Model group decreased, while the above transduction pathways in the VA, VD, and VA + VD groups increased.

### 4.4 Effects of VA and VD on the intestinal flora of TS rats

**Figure 5:**
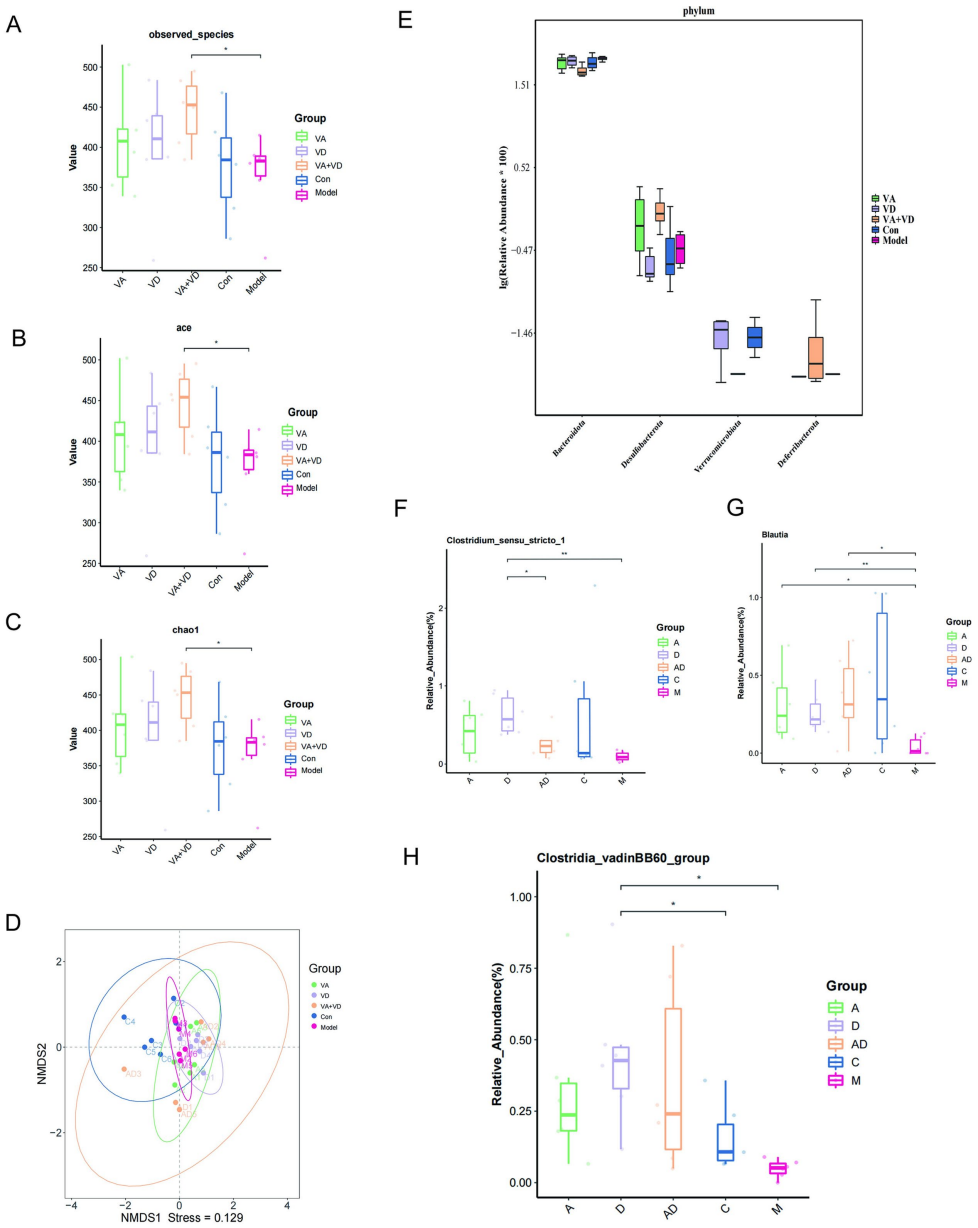
Changes in fecal bacterial composition in TS rats at 10 weeks. Bacterial *α* diversity **(A–C)** was determined using the observed-species index, ace index, and Chao 1 index; *β* diversity **(D)** based on NMDS; different phylum composition **(E)** for each group and based on Kruskal–Wallis differential analysis **(F–H)**. (Values are means ± SEMs; *n* = 6,*p<0.05, **p<0.01).

A close relationship between TS and intestinal flora has been reported in the literature. The effects of VA and VD on the structure of intestinal flora in TS rats were detected by 16 s rRNA gene sequencing analysis. Through the relative abundance analysis, the results showed that the groups showed significant differences, and there was no significant difference between the vitamin and Con groups, indicating that vitamin A and D supplementation could attenuate IDPN-induced intestinal microbiota dysbiosis in TS rats. The species diversity and richness of the intestinal flora community were analyzed by the *α*-diversity analysis method, the observed-species index was used to analyze the diversity of the intestinal flora community, and the Chao1 index and ace index were used to evaluate the richness of the intestinal flora community. The observed-species index of the VA + VD group was significantly higher than that of the Model group (*p* < 0.05). Still, the VA, VD, and Con groups had an increasing trend compared with the Model group (Figure 5A). For the Chao1 index and ace index, there were similar differences between groups, that is, IDPN treatment decreased the richness of the enterobacterial community in TS rats, but vitamins A and D prevented this decrease (Figures 5B,C). This result indicated that vitamins A and D ameliorated IDPN-induced changes in the species diversity and richness of intestinal flora in TS rats. Based on the *β* diversity of NMDS, we found that the dispersion of samples among different groups was obvious, especially between the Model and Con groups, indicating that significant changes in the composition of the gut microbiota were in the TS rat model (Figure 5D). The gut microbiota composition after vitamin A and D treatment was partially similar to that of the Con group, indicating that after vitamin A and D supplementation, the gut microbiota composition was regulated and tended to approach the Con group. Based on the analysis of Kruskal–Wallis flora differences, at the phylum level, significant differences were found between the vitamin and model groups, mainly for Bacteroidota, Desulfobacterota, Verrucomicrobiota, and Deferribacterota (Figure 5E). Significant differences at the genus level were Clostridium _sensu _stricto _1 and Blautia, Clostridia _vadinBB60 _group (Figures 5F–H).

### 4.5 Association analysis of intestinal flora and metabolomics

**Figure 6:**
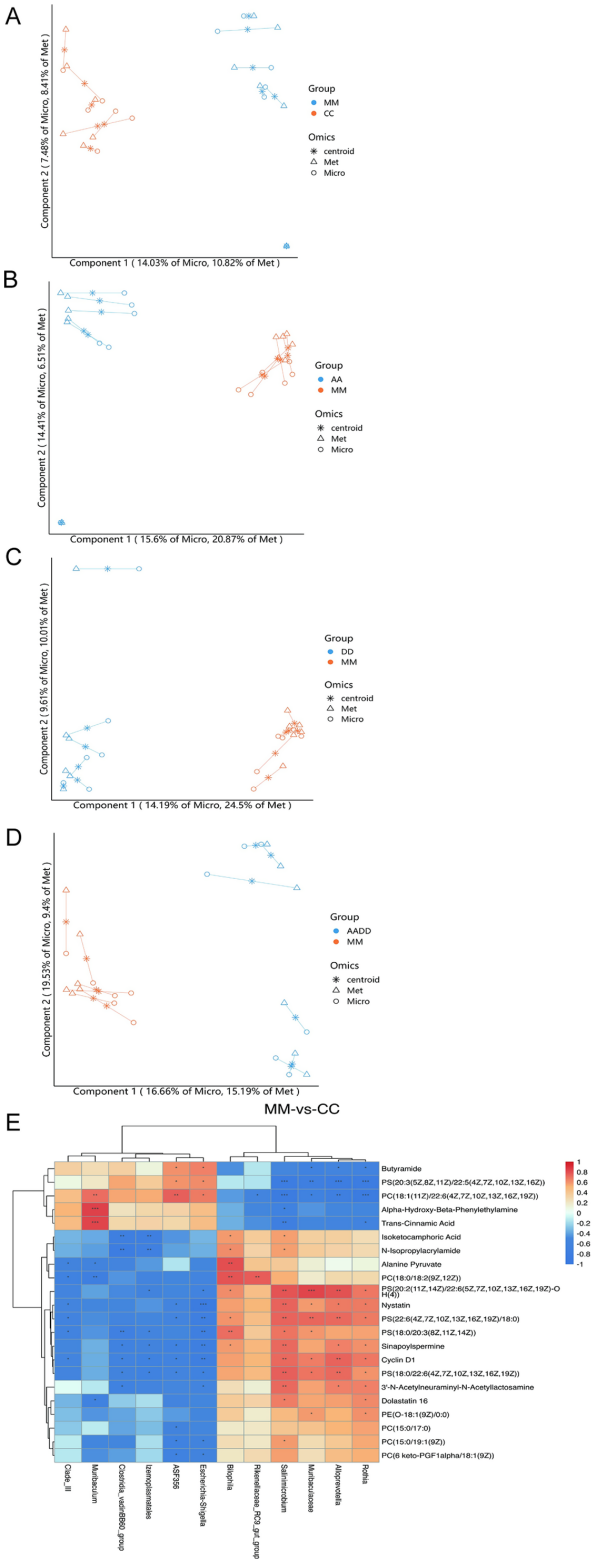
Correlation of the fecal microbiome with striatal metabolites. For the metabolites and the microbial genus, which groups are Model and Con (MM vs. CC), VA and Model (AA vs. MM), VD, and Model (DD vs. MM), VA + VD and Model (AADD vs. MM). Spearman’s rank correlation coefficient was> 0.5, *p* < 0.05 **(A–D)**. Spearman correlation heat map between gut microbiota and striatal metabolites. Statistically significant correlation between the two groups that Model and Con (MM vs. CC) was tested using the Pearson’s correlation coefficient, *p* < 0.05 **(E)**. (Values are means ± SEMs; *n* = 6).

To investigate the potential correlation between gut microbiota composition and differential metabolites of the striatum in vitamin A, D, and TS rats, we performed a Spearman correlation analysis. As shown in the relationship between striatal metabolites and microbial communities at the genus level, the samples were clustered by grouping (Figures 6A–D). The Model group was compared to the Con group (MM vs. CC), and the selected strains showed a very significant correlation with 22 hypothalamic metabolites. Specifically, there is a negative correlation between Alloprevotella, Rothia, Muribaculaceae, Salinimicrobium, Rikenellaceae _ RC9 _ gut _ group, Bilophila, and Butyramide, PS (20: 3 (5Z, 8Z, 11Z)/22: 5 (4Z, 7Z, 10Z, 13Z, 16Z)), PC (18: 1 (11Z)/22: 6 (4Z, 7Z, 10Z, 13Z, 16Z, 19Z)), alpha-hydroxy-beta-phenylethylamine, and trans-cinnamic acid, but positively correlated with the other 17 products. In contrast, the correlation between the other 6 bacteria and striatum metabolites was opposite (Figure 6E).

## 5 Discussion

In this study, a TS rat model induced by IDPN was used to explore the role of VA and VD in alleviating TS-related behaviors. Our results showed that supplementation with VA and VD significantly reduced tic-like behaviors in TS rats. This effect may be attributed to changes in neurotransmitter activity and the modulation of intestinal microecology by these vitamins. IDPN-induced TS rats exhibited increased HTR, stereotyped behaviors, and reduced body weight. After VA and VD supplementation, these behavioral symptoms were reduced, and the body weight increased, suggesting that VA and VD have neuroprotective effects and support growth and development. Research indicates that the treatment of VA and VD can help mitigate the incidence of TS during clinical treatment. A randomized clinical trial demonstrated that after 3 months of VD supplementation in 120 children with TS, there were improvements in the total score on the Yale Global Tic Severity Scale (YGTSS), including significant improvements in motor tics and vocal tics, without any reported adverse effects (26). It has also been shown that VA levels in children with TS are significantly lower than in healthy children (27).

Given that the dose of VD necessary to protect the nervous system is significantly higher than that required for regulating calcium and phosphorus metabolism and maintaining skeletal health, potential side effects, such as hypercalcemia, may arise (28). In our study, we measured the 25-OH-D₃ content and related calcium metabolism indicators, including CA, P, and ALP. The results indicated an increase in 25-OH-D₃ levels in both the VD and VA + VD groups, while other indicators remained within normal ranges.

An analysis of differential metabolites in the striatum across all experimental groups revealed significant variations in fatty acids, amino acids, and depsipeptides. Notably, the L-lysine content decreased in the TS rats but increased following VA and VD supplementation. L-Lysine is known to supplement essential amino acids in the human body and is associated with immune function and the repair of damaged nerve tissues (29). It influences neurotransmission, synapse formation, membrane signaling pathways, and neuroinflammation in the brain, thereby linking it to various central nervous system disorders, including autism spectrum disorder, Parkinson’s disease, and Alzheimer’s disease (30). Clinical studies have demonstrated that lysine supplementation can significantly reduce anxiety and enhance stress responses (31). Therefore, we hypothesize that VA and VD supplementation may positively influence neurotransmission and synaptic function in TS rats by modulating L-lysine levels, subsequently improving their twitching behaviors.

The retrograde endocannabinoid pathway—a key modulator of synaptic transmission and dopaminergic circuits—emerges as a critical metabolic pathway in tic disorders. This system comprises cannabinoid receptors (CBRs), endogenous ligands (such as 2-arachidonoylglycerol and arachidonamide), and its metabolic enzymes. Cannabis plant: delta-α9-tetrahydrocannabinol (THC) and cannabidiol (CBD) may attenuate dopamine transmission via CB2R/TRPV1 activation in nigrostriatal neurons, potentially explaining their motor-suppressive effects (32, 33). A clinical randomized controlled trial involving patients aged 18 to 60 with TS demonstrated a significant reduction in tic symptoms, along with decreased obsessive-compulsive and anxiety symptoms in the cannabidiol group (34). Furthermore, a follow-up study of 42 TS patients using medical marijuana revealed that 38 participants experienced reduced tic severity, improved sleep quality, and enhanced mood (35). Our findings suggest that VA and VD supplementation elevates phosphatidylserine levels, activating endocannabinoid retrograde signaling to modulate dopaminergic function—a potential therapeutic strategy for TS.

Studies have found that intestinal microorganisms and their metabolites play a crucial role in maintaining physiological processes, including the integrity of the intestinal barrier, enhancing intestinal endocrine function, supporting immune system formation, and regulating nervous system activities (36, 37). Clinical observations indicate that TS patients frequently exhibit gastrointestinal symptoms, such as constipation and indigestion, which are often linked to imbalances in intestinal flora (38). Notably, a reported 8-week single-dose fecal microbiota transplantation (FMT) treatment led to an increase in beneficial intestinal bacteria in TS patients, resulting in a significant improvement in tic symptoms and a marked decrease in YGTSS scores (39). Another animal study found that *Lactobacillus plantarum* PS128 administration for FMT treatment led to reduced tic-like behaviors and improved intracerebral dopamine and norepinephrine levels in TS rats (40).

The TS model group exhibited reduced gut microbiota diversity (richness and evenness) alongside increased tic-like behaviors. Moreover, specific microbial populations, such as Lactobacillus, Clostridia_vadinBB60_group, and Blautia, were depleted, while Rikenellaceae_RC9_gut_group was enriched. VA and VD supplementation restored microbial balance, notably boosting Lactobacillus—a genus known to modulate immunity via metabolite-driven suppression of pro-inflammatory cytokines and induction of anti-inflammatory responses (41). Previous studies have indicated that children with TS exhibit autoimmune dysfunction and a state of cellular immune activation, with inflammatory cytokines contributing to TS pathology (42). Thus, we propose that VA and VD may exert immunomodulatory effects by regulating intestinal flora balancing, thereby alleviating tic behaviors in TS rats.

Intestinal flora may regulate the body’s physiological state by participating in metabolism. Our study identified significant changes in the intestinal microorganisms of TS rats, with key microorganisms (Alloprevotella, Rothia, Muribaculaceae, Salinimicrobium, Rikenellaceae_RC9_gut_group, Bilophila) exhibiting a significant correlation with their brain metabolites. These observations have been corroborated by extensive research addressing the mechanisms underlying neurodevelopmental disorders. Specifically, the Muribaculaceae family has been implicated in the pathophysiological processes associated with Parkinson’s disease. Alloprevotella contributes to host health by producing short-chain fatty acids (SCFAs), participating in bile acid metabolism, enhancing resistance to pathogen colonization, and playing roles in various brain diseases. For example, SCFA levels are reportedly diminished in patients with Alzheimer’s and Parkinson’s diseases (43, 44). Alloprevotella metabolizes to produce acetate, which Rothia subsequently utilizes to synthesize butyrate—an essential energy source for intestinal epithelial cells that is also involved in inhibitory signaling pathways for pro-inflammatory cytokines (45). This administration offers a potential novel therapeutic option compared to conventional therapies; however, its long-term safety and efficacy still need further investigation. Moreover, our study has some limitations that should be addressed. Given the higher incidence of TS in men compared to women, we exclusively established and explored TS models in male mice without examining changes in females. However, the role of gender differences in TS cannot be ignored. It has been suggested that sex hormones (such as testosterone and estrogen) may affect the pathological process of TS by regulating the dopaminergic neurotransmission (46, 47). The effect of gender on the composition and function of the gut microbiota may also contribute to differences in TS symptoms and responses to treatment. Future studies should further investigate the specific role of vitamins in TS.

## 6 Conclusion

Our study demonstrates that VA and VD supplementation alleviate TS-related behaviors in rats by modulating intestinal flora, regulating striatal metabolism, and enhancing neuroprotective metabolites. VD, in particular, plays a dominant role in TS treatment. These findings further support the microbe–gut–brain axis hypothesis in TS and offer new insights into the clinical management of TS.

## Data Availability

The datasets presented in this study can be found in online repositories. The names of the repository/repositories and accession number(s) can be found in the article/supplementary material.
